# Diversity of electroencephalographic patterns during propofol-induced burst suppression

**DOI:** 10.3389/fnsys.2023.1172856

**Published:** 2023-06-15

**Authors:** Keith G. Jones, Carter Lybbert, Matthew J. Euler, Jason Huang, Seth Lunt, Sindhu V. Richards, Jacob E. Jessop, Adam Larson, David H. Odell, Kai Kuck, Scott C. Tadler, Brian J. Mickey

**Affiliations:** ^1^Interdepartmental Program in Neuroscience, The University of Utah, Salt Lake City, UT, United States; ^2^Department of Psychiatry, Huntsman Mental Health Institute, The University of Utah, Salt Lake City, UT, United States; ^3^Department of Biomedical Engineering, The University of Utah, Salt Lake City, UT, United States; ^4^Department of Anesthesiology, The University of Utah, Salt Lake City, UT, United States; ^5^Department of Psychology, The University of Utah, Salt Lake City, UT, United States; ^6^Department of Neurology, The University of Utah, Salt Lake City, UT, United States

**Keywords:** propofol, electroencephalograph (EEG), burst suppression, anesthesia, depression

## Abstract

Burst suppression is a brain state consisting of high-amplitude electrical activity alternating with periods of quieter suppression that can be brought about by disease or by certain anesthetics. Although burst suppression has been studied for decades, few studies have investigated the diverse manifestations of this state within and between human subjects. As part of a clinical trial examining the antidepressant effects of propofol, we gathered burst suppression electroencephalographic (EEG) data from 114 propofol infusions across 21 human subjects with treatment-resistant depression. This data was examined with the objective of describing and quantifying electrical signal diversity. We observed three types of EEG burst activity: canonical broadband bursts (as frequently described in the literature), spindles (narrow-band oscillations reminiscent of sleep spindles), and a new feature that we call low-frequency bursts (LFBs), which are brief deflections of mainly sub-3-Hz power. These three features were distinct in both the time and frequency domains and their occurrence differed significantly across subjects, with some subjects showing many LFBs or spindles and others showing very few. Spectral-power makeup of each feature was also significantly different across subjects. In a subset of nine participants with high-density EEG recordings, we noted that each feature had a unique spatial pattern of amplitude and polarity when measured across the scalp. Finally, we observed that the Bispectral Index Monitor, a commonly used clinical EEG monitor, does not account for the diversity of EEG features when processing the burst suppression state. Overall, this study describes and quantifies variation in the burst suppression EEG state across subjects and repeated infusions of propofol. These findings have implications for the understanding of brain activity under anesthesia and for individualized dosing of anesthetic drugs.

## 1. Introduction

Burst suppression is an electroencephalogram (EEG) pattern consisting of bursts of activity alternating with near-isoelectric periods of suppression. These bursts are typically high-amplitude and quasi-periodic, lasting several seconds and occurring multiple times per minute (Swank and Watson, 1949). Burst suppression is seen in the human brain during pathological states such as coma (Brown et al., 2010), hypothermia (Brandon Westover et al., 2015), cerebral ischemia (Hofmeijer et al., 2014), and early infantile epileptic encephalopathy (Ohtahara syndrome) (Saneto and Sotero De Menezes, 2007). It can also occur during the use of certain anesthetics, specifically GABAergic drugs that lower the cerebral metabolic rate of oxygen (Hirsch and Taylor, 2010; Ching et al., 2012). Though burst suppression is often seen as an unwanted outcome, the literature on the prognosis of patients who experience this state is mixed (Ma and Bebawy, 2022). Burst suppression has been associated with increased mortality (Watson et al., 2008) and post-operative delirium (Andresen et al., 2014; Fritz et al., 2016) but also with protection against post-operative cognitive dysfunction in the elderly (Deiner et al., 2015). Burst suppression may be intentionally induced to reduce neural metabolic demand, such as when treating refractory status epilepticus (Parviainen et al., 2006; Phabphal et al., 2018) or during neurosurgical procedures (Engelhard and Werner, 2006; Schlünzen et al., 2012). Furthermore, preliminary evidence suggests that a brief period of burst suppression may have antidepressant effects (Langer et al., 1995; Weeks et al., 2013; Mickey et al., 2018).

Recent work has highlighted the complexities of burst-suppression states, demonstrating that burst suppression is not a unitary phenomenon. First, burst suppression does not occur consistently across all populations; specifically, older subjects (Besch et al., 2011; Purdon et al., 2015) and those with decreased frontal alpha power during anesthesia (Shao et al., 2020) seem to be more vulnerable to this state. Secondly, there is variance in the burst suppression waveform depending on its cause. Isoflurane or sevoflurane anesthesia tends to create bursts that are higher in amplitude, but lower in relative alpha power, when compared to propofol bursts (Akrawi et al., 1996; Fleischmann et al., 2018). Cerebral ischemia can lead to bursts that are short in duration and nearly identical in appearance (Hofmeijer et al., 2014). Also, burst suppression may not be a simple alternation between binary states. Several groups have examined propofol anesthesia in humans and reported spindles, features consisting of 13–17 Hz activity lasting for one to multiple seconds that are reminiscent of sleep spindles and morphologically distinct from bursts (Särkelä et al., 2002; Huotari et al., 2004; Ferenets et al., 2006; Wolter et al., 2006). Huotari et al. (2004) noted that spindles often follow bursts but also occur spontaneously throughout burst suppression and even during complete suppression, potentially implying a unique generator such as the thalamus. Other researchers have described burst suppression with large spikes and burst-suppression-like patterns with higher amplitude suppressions (Niedermeyer et al., 1999). There is also evidence that characteristics of the bursts themselves may vary significantly; burst activity in older patients tends to have a lower amplitude, decreased absolute 1–15 Hz power, increased relative 15 + Hz power, and increased permutation entropy when compared to burst suppression in younger patients (Kratzer et al., 2020). Burst amplitude and frequency content have also been correlated with positive clinical outcomes in IV anesthetic therapy for refractory status epilepticus (Johnson et al., 2016). Finally, the burst suppression state is not spatially homogenous throughout the brain. Previous research has shown that bursts can occur both locally and globally across the cortex (Lewis et al., 2013; An et al., 2015) and that burst onset and spectral makeup changes with location (Lewis et al., 2013).

The existence of different types of burst activity with different morphologies has implications for accurate clinical monitoring and dosing during anesthesia. Commonly used clinical EEG monitors, such as the Bispectral Index (BIS) Monitor, evaluate burst suppression as a binary state (Chemali et al., 2013) and do not distinguish differences in burst activity during or across recordings. These monitors give a summary of frontal EEG that may not be indicative of the full range of electrical activity and may underestimate total suppression when compared to visual analysis (Muhlhofer et al., 2017). Despite the known complexities of burst suppression, there has been limited research into how EEG activity during burst suppression varies across patients and over repeated bouts of anesthesia.

Here, we explore the variation in EEG burst suppression between subjects and across multiple sessions of high-dose propofol infusions in a cohort with treatment-resistant depression. We distinguish three types of EEG activity in humans during deep propofol anesthesia: canonical broadband bursts (CBBs) as described in previous literature, spindles, and a new type of activity, hereafter referred to as low-frequency bursts (LFBs). Our objectives were (1) to describe the variability of each of these EEG features across subjects and infusion sessions, (2) to determine how the three types of activity are spatially and temporally related to each other, and (3) to evaluate the impact of these diverse features on the accuracy of clinical EEG monitoring.

## 2. Materials and methods

As part of a clinical trial (NCT03684447), 24 participants with treatment-resistant depression were randomized to receive 6 infusions of high- or low-dose propofol anesthesia to examine the drug’s antidepressant effects. Those subjects who were randomized to low-dose propofol and did not experience an antidepressant response could choose to receive 6 additional open-label high-dose infusions. A total of 21 participants across both arms of the study received high-dose infusions and had burst suppression EEG available for analysis. These subjects were otherwise healthy, ranged in age from 21 to 57 years, and consisted of 10 males and 11 females (Table 1). Exclusionary criteria included a body mass index >40, a primary psychiatric diagnosis other than depression, age outside the range of 18–65, or contraindication to anesthesia. Participants received the 6 propofol infusions over a span of 2 weeks. Subjects were instructed to continue their psychiatric medications unchanged, and twenty out of twenty-one subjects were on a mixture of antidepressants and augmentation medications (Supplementary Table 1). Ondansetron (4 mg) and lidocaine (30 mg) were given as pre-medication before each infusion. Pre-medication changes included: two subjects did not receive ondansetron; one subject did not receive lidocaine; seven subjects received ketorolac; two subjects administered albuterol inhalers; one subject received dexamethasone; and one subject received sodium citrate and pantoprazole. During the infusions, two subjects received ephedrine, and one subject received succinylcholine. There was no clear relationship between any of these medication changes and the EEG signal. Propofol was dosed to an EEG target window of 70–90% burst suppression ratio (BSR), as measured by the BIS Monitor, for 12–15 min. Anesthesia was induced with a propofol bolus to quickly achieve burst suppression, and this state was maintained with a propofol infusion using a Medfusion infusion pump. No additional anesthetics or opioids were used. Total doses for individual treatments ranged from 400 to 1200 mg.

**TABLE 1:** Demographics and anesthesia parameters for each subject.

Subject	Age	Sex	Average BIS BSR	Average C_e_
1	51	F	76.2 (2.9)	4.7 (0.9)
2	39.9	F	62.8 (6.1)	6.6 (0.2)
3	33.8	M	72.9 (3.1)	6.6 (0.1)
4	49.2	F	67.5 (7.5)	5.4 (1.4)
5	51.3	F	57.8 (6.7)	5.3 (0.3)
6	48.3	F	56.3 (14.1)	6.9 (0.6)
7	30.8	F	69.3 (4.5)	7.3 (1.4)
8	29.3	F	66.4 (8.5)	7.2 (0.7)
9	32.6	M	71.3 (2.6)	7.5 (0.6)
10	39.2	F	70.7 (5.4)	6.3 (0.8)
11	29.4	F	76.1 (3.2)	4.1 (0.4)
12	36.1	M	70.8 (4.4)	8.5 (0.6)
13	49.2	M	77.9 (2.3)	5.9 (0.6)
14	37	M	71.8 (3.9)	6.6 (0.3)
15	57.6	M	75.6 (2.3)	6.1 (0.4)
16	40.5	M	70.8 (4.5)	6.1 (0.5)
17	35.1	M	78.1 (4.8)	5.9 (0.5)
18	52.3	F	71.8 (6.3)	4.2 (0.5)
19	39.3	M	74.5 (5.6)	7.8 (1.7)
20	21.5	F	65.5 (5.6)	6.3 (0.4)
21	24.5	M	53.2 (6.6)	6.5 (0.7)

Mean (standard deviation). Sex indicates sex assigned at birth.

### 2.1. EEG acquisition

Electroencephalographic data was acquired using a BIS Monitor (BIS VISTA Monitoring System, Aspect Medical Systems, Newton, MA, USA) with a BIS Quatro sensor (Medtronic, Dublin, Ireland) on the left forehead. The raw data consisted of two channels of data (sampling rate, 128 Hz) with a reference channel on the left forehead, and it was exported from the BIS Monitor and imported for analysis via MATLAB (version R2020b), where analysis was performed on the first channel. Raw EEG amplitude was converted into microvolts using a coefficient of 0.05 (Connor, 2022). Each participant underwent a 5-min resting baseline recording, and data was acquired from pre-bolus until spontaneous waking during recovery. EEG data was acquired for a total of 114 infusions across 21 subjects. To characterize variation across scalp locations, 9 participants also had one recording of 64-channel EEG (sampling rate, 500 Hz) using an actiCHamp amplifier and an actiCAP slim electrode system (BrainVision, Garner, NC, USA).

### 2.2. EEG processing

Electroencephalographic data from the BIS Monitor was imported into MATLAB using a custom script. Each recording was visually inspected, and large artifacts (such as those from airway manipulation or BIS Monitor ground checks), distinguished by their high amplitude compared to the surrounding activity, were manually marked for removal. Raw BIS Monitor data showed characteristics of low-pass filtering with a cut-off of ∼45 Hz, as the power spectrum declined by approximately 15 dB from 45 to 49 Hz. During software processing, the data was first high-pass filtered with a Butterworth filter designed using the following parameters: passband = 0.5 Hz, stopband = 0.1 Hz, stopband attenuation = 3 dB, passband ripple = 1 dB (“fdesign” and “filter” functions, DSP System Toolbox). It was then low-pass filtered using a similar design with a passband of 43 Hz and a stopband of 45 Hz. These parameters were chosen in order to filter out the DC offset while retaining the morphology of all EEG features (see Supplementary Figure 1 for a comparison of pre- and post-filtered data). Särkelä et al. (2002) note that there are no burst characteristics above 47 Hz during human propofol anesthesia, which we also confirmed with our 64-channel EEG recordings.

The 64-channel EEG data (reference Cz) was loaded into MATLAB for analysis using EEGLAB [v2022.0 (Delorme and Makeig, 2004)] and filtered with a basic finite impulse response filter to include frequencies between 0.1 and 45 Hz. This removed any DC baseline and allowed the data to be directly compared to BIS Monitor recordings. Any overly noisy channels were removed and interpolated. Data was visually inspected to evaluate the presence or absence of different features and their cortical locations. The Fz channel was then used for segmentation as explained below. For the one subject who had consistent LFBs in this data set, multiple channels across the scalp were used for segmentation and compared in order to capture all of the localized features. The data was then transformed via surface Laplacian into a reference-free set of current source densities using the CSD toolbox (version 1.1) (Kayser and Tenke, 2006).

### 2.3. Burst segmentation

Electroencephalographic features were identified using a custom MATLAB script. First, a continuous wavelet transform (“cwt” function, Wavelet toolbox) was used to calculate power at different frequencies. The continuous wavelet transform, rather than short-time Fourier transform, was used to more accurately capture the spectral properties of a continuously changing signal. By default, the cwt function in MATLAB uses the analytic Morse wavelet (Olhede and Walden, 2002) with a time-bandwidth product of 60, a symmetry parameter of 3, and includes L1 normalization. These parameters produce a wavelet that is highly symmetric in both the time and frequency domains and ideal for the analysis of abruptly changing signals (Lilly and Olhede, 2009). Additional analytic wavelets were tested (Morlet, bump), and Morse was the best option to capture both high- and low-frequency patterns (see Supplementary Figure 1 for comparison). Voices per octave were set to 30 for increased frequency resolution. Using a 1-s sliding window, canonical broadband bursts were identified as areas of activity that had high total power above 3 Hz. Low-frequency bursts were identified as sections of data with high power below 3 Hz but lower power elsewhere. Spindles were identified as sections of data with high power between 13 and 17 Hz but low power elsewhere. Suppressions were defined as epochs that did not contain a CBB, LFB, or spindle; thus, this method was more akin to “burst detection” than the “suppression detection” used by the BIS Monitor and other common clinical EEG monitors. Only activity at BSR > 30% was included in order to most clearly differentiate individual bursts and suppressions. Below a BSR of 30%, suppressed periods shorten to the point where individual features cannot be identified or quantified. The emergence of large delta waves, a characteristic of the period after propofol offset but before waking, further compounds this issue. After the EEG was segmented into these three types of activity, several parameters were calculated for each. These included duration, peak-to-peak amplitude, and frequency content. A custom BSR (denoted CBSR) was calculated by including only canonical broadband bursts and excluding low-frequency bursts and spindles.

### 2.4. Effect site concentration modeling

A population-based, three-compartment, pharmacokinetic/pharmacodynamic model was used to estimate the propofol effect site concentration (C_*e*_) for each infusion by taking into account each participant’s age, sex, weight, and height (Eleveld et al., 2018; Vellinga et al., 2021). The second-by-second dosing of propofol that each participant received was entered into the model, which returned an estimated second-by-second effect site concentration for each treatment of each participant (Eleveld et al., 2018).

### 2.5. Statistical analysis

Statistical analysis was conducted using the R statistical software (version 4.2.2). Average feature parameters were calculated using a weighted average, with each infusion and subject being weighted equally, to balance the unequal occurrence per infusion. To evaluate variation in the occurrence of EEG features between subjects, ANOVA models were created (“aov” function, base R) with subject number as an independent factor and number of features per treatment as a dependent variable. Kruskal-Wallis tests (“kruskal.test” function, base R) were using to evaluate the number of LFBs and spindles, as they were not normally distributed across subjects. Normality was tested using the Shapiro-Wilks test (“shapiro.test” function, base R). Effect size for these tests is presented as η^2^ (“kruskal_effect” function, “rstatix” package, version 0.7.0). To explain the variation in feature occurrence, linear mixed models were used (“lmer” function in “lme4” package, version 1.1-29). Fixed effects included treatment number, infusion duration, average CBSR, average EEG feature duration, age, and sex. Subject number was included as a random intercept. A factor was considered statistically significant if its *p*-value was <0.05 (“Anova” function in “car” package, version 3.0-13). Semi-partial *R*^2^ values were calculated using the “r2beta” function (“r2glmm” package, version 0.1.2). These were chosen as the most accurate way to convey effect sizes in linear mixed models and represent the amount of variance explained by a fixed effect when adjusted for all other effects in the model (Jaeger et al., 2017). MANOVA models were used to compare feature spectral power between subjects, as the relative percentages of delta, theta, alpha, and beta power are not independent (“manova” function, base R).

To analyze timing between EEG features, cross- and auto-correlations were performed using MATLAB (“crosscorr” and “autocorr” functions, base MATLAB). For each of the three features, a binary time series was created by concatenating data from all infusions for all subjects together and setting the starting time of each feature occurrence to 1. The time series was downsampled to 10 Hz, and a cross correlation was run using 0.1 s lags out to 10 s for CBBs and spindles, CBBs and LFBs, and spindles and LFBs. Confidence intervals were defined to include 99% of values around the null mean of 0.

To evaluate the relationship between feature type and onset time, linear mixed models were created in R. Feature type and infusion number were included as fixed effects, and subject number was included as a random intercept. Similar models were used to analyze the relationship between feature type, C_*e*_, and infusion session number. To compare relative bandpower of EEG features across subjects, average BSR, average EEG feature duration, age, and sex were included as fixed effects, and subject number was included as a random intercept.

## 3. Results

### 3.1. Types of activity

**FIGURE 1:**
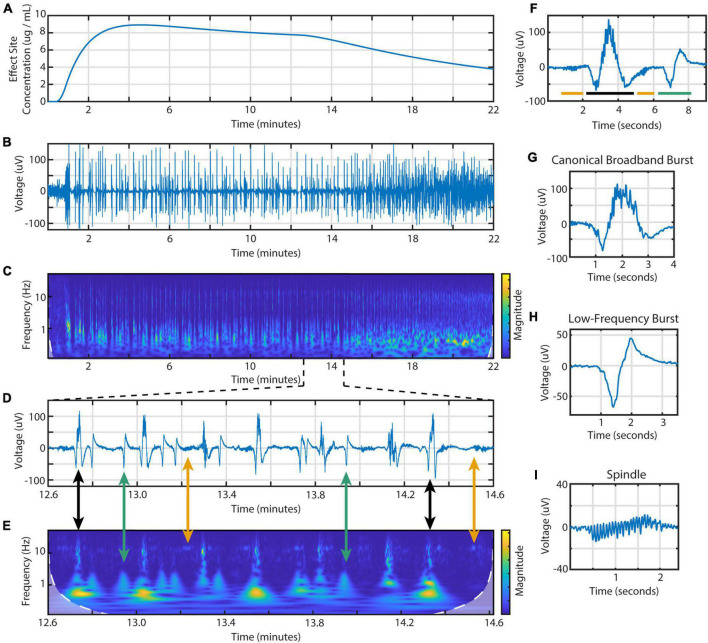
Time- and frequency-domain representations of burst suppression during an individual propofol infusion session. Data is from the BIS Monitor EEG of subject 2, infusion 4. **(A)** The estimated effect site concentration, **(B)** raw EEG and **(C)** scalogram from 22 min of a propofol infusion. **(D)** A zoomed in section showing the three types of EEG activity and **(E)** scalogram. Black arrows indicate canonical broadband bursts; green, low-frequency bursts; yellow, spindles. **(F)** Individual examples of all three features including a **(G)** canonical broadband burst, **(H)** low-frequency burst, and **(I)** spindle.

From the BIS frontal EEG data, we identified three different types of EEG activity during burst suppression (Figure 1). These waveforms are distinct in both the time and frequency domains. First is the canonical broadband burst (CBB) as frequently described in the literature: a multi-second waveform that consists of a large, low-frequency positive deflection overlaid with 10 Hz activity. Second, we found a distinctive type of electrical activity that we call low-frequency bursts (LFBs). Shorter than CBBs, these biphasic deflections consist mostly of low-frequency activity with little-to-no 10 Hz power. Finally, we saw spindles: periods of low amplitude activity with consistent, narrowband waves centered from 13 to 17 Hz.

Using these time and frequency characteristics, we developed a custom MATLAB script to extract all features from the 114 high dose infusions available. This amounted to a total of 7017 CBBs, 1437 LFBs, and 2835 spindles. In the time domain, these three types of activity were differentiated by their morphology, duration, and amplitude (Table 2). The average canonical broadband burst was 3.58 ± 1.01 (mean ± SD) seconds in length and 128 ± 35 μV in peak-to-peak amplitude. Low-frequency bursts averaged 1.78 ± 0.30 s in length and 63.9 ± 14.5 μV in peak-to-peak amplitude. Finally, spindles averaged 1.12 ± 0.30 s in length and 24.8 ± 9.3 μV in peak-to-peak amplitude. In the frequency domain, each type of activity was also distinct. On a scalogram calculated using continuous wavelet transform (Figures 1C, E), canonical broadband bursts can be seen as large, high amplitude spikes with broadband power from 1 to 12 Hz. LFBs are similar to CBBs but have slightly lower power and only consist of the base (sub 3 Hz) frequencies. Finally, spindles can be seen as areas of increased power around 14 Hz, often including a smaller peak one octave higher at 28 Hz. The average ± SD peak spindle frequency across all subjects and infusions was 14.4 ± 1.13 Hz.

**TABLE 2:** Summary table of the EEG activity for each study participant weighted equally for each infusion.

Subject number	Mean CBBs per minute	Mean CBB duration (sec)	Mean CBB peak-to-peak amplitude (uV)	Mean LFBs per minute	Mean LFB duration (sec)	Mean LFB peak-to-peak amplitude (uV)	Mean spindles per minute	Mean spindle duration (sec)	Mean spindle peak-to-peak amplitude (uV)
1	2.2 (0.6)	3.6 (0.9)	126.2 (42.2)	1.2 (0.2)	1.8 (0.3)	66.8 (13.1)	1.2 (0.5)	1.4 (0.4)	24.5 (11.5)
2	3.3 (1)	3.6 (1)	163.3 (44.5)	2.7 (0.6)	1.9 (0.3)	87.6 (18.2)	4.1 (1.2)	1.3 (0.5)	25.3 (13)
3	4.5 (0.5)	3.3 (0.8)	118.6 (28.8)	0.2 (0.1)	2.2 (0.7)	84.4 (18)	0.8 (0.2)	1 (0.2)	28.2 (9.8)
4	4.7 (0.8)	3.2 (0.8)	106.6 (25.3)	0.2 (0.2)	1.7 (0.1)	58.4 (9.1)	2.3 (0.9)	1.3 (0.3)	22.6 (7.1)
5	4.2 (1.8)	3 (0.7)	92.4 (30.3)	2.8 (0.8)	1.8 (0.3)	57.3 (11.7)	1.4 (0.8)	1.1 (0.2)	23.6 (7.6)
6	5.9 (1.8)	3.6 (1)	111 (24.6)	0.7 (0.2)	1.7 (0.2)	67.1 (14.4)	0.3 (0.2)	0.9 (0.2)	22.5 (8.8)
7	4 (0.8)	3.7 (1.3)	150.5 (40.7)	0.1 (0.1)	1.7 (0.1)	65.7 (15.7)	1.3 (1)	1.1 (0.3)	19.8 (5)
8	2.4 (0.9)	3.7 (0.8)	191.7 (45.2)	0.8 (0.1)	1.9 (0.3)	73.9 (13.8)	1.8 (0.8)	1.2 (0.4)	21.7 (6.9)
9	3.9 (0.7)	4 (1.6)	152.4 (40.2)	0.2 (0.1)	1.8 (0.2)	64.6 (14.6)	1.9 (0.4)	1.2 (0.3)	29.7 (13.4)
10	4.3 (1)	3.7 (1.1)	135.5 (38)	0.4 (0.2)	1.9 (0.4)	73.2 (17.2)	0.9 (0.6)	1.2 (0.3)	20 (5.1)
11	3.3 (0.6)	3.4 (0.9)	130.9 (35.6)	0.3 (0.3)	1.6 (0.2)	65.4 (17.3)	1.1 (0.3)	1 (0.2)	28.4 (10.9)
12	3.1 (0.4)	3.2 (1)	133.8 (52.9)	0.9 (0.4)	1.8 (0.3)	62.2 (18.4)	2.4 (1.1)	1.3 (0.4)	22.1 (7.1)
13	3 (0.5)	3.7 (1)	108.4 (25.7)	0 (0.1)	1.7 (0.2)	57.3 (9.8)	1.7 (1.3)	1 (0.3)	21.9 (6.5)
14	3.9 (0.7)	3.3 (1)	97.5 (33.2)	0.6 (0.3)	1.7 (0.2)	52.8 (10.2)	0.5 (0.9)	1 (0.2)	23.5 (5.1)
15	4.3 (0.4)	3 (1.1)	76.2 (20.4)	0.3 (0.1)	1.8 (0.4)	61.2 (13.2)	1.2 (0.3)	1.1 (0.3)	25.9 (12.3)
16	3.2 (0.5)	3.7 (1.1)	105.1 (27.8)	0.2 (0.1)	1.8 (0.3)	56 (13.5)	0.1 (0.2)	1 (0.2)	35.4 (9.5)
17	2.6 (0.8)	4.6 (1.2)	141.1 (25.8)	0.1 (0.1)	1.7 (0.3)	60.2 (15)	0.2 (0.3)	1 (0.3)	19.3 (12.8)
18	3.6 (0.7)	3.7 (1.1)	88.2 (25.1)	0.3 (0.2)	1.8 (0.5)	59.3 (11)	0.5 (0.3)	0.9 (0.2)	25.4 (10.5)
19	2.8 (0.8)	3.7 (1)	148.1 (32.8)	0.3 (0.1)	1.7 (0.2)	50.2 (12.8)	0.8 (0.4)	1.1 (0.2)	21 (7.9)
20	3.3 (0.7)	3.7 (1.1)	171.1 (52.5)	1.6 (0.3)	1.8 (0.3)	63.9 (14.2)	0.8 (0.3)	0.9 (0.2)	30.5 (12.4)
21	3.6 (0.6)	3.9 (0.9)	146.7 (34.9)	0.2 (0.2)	1.6 (0.3)	54.7 (22.4)	4.8 (1.8)	1.5 (0.6)	30.5 (12.3)

Mean (standard deviation).

**FIGURE 2:**
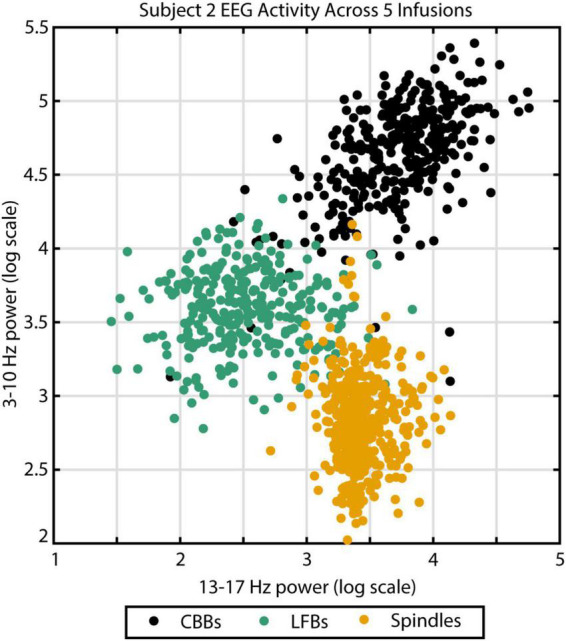
The three different EEG burst-suppression features can be differentiated by their frequency parameters. Canonical broadband bursts (CBBs) exhibit the highest power in the 3–10 Hz range, spindles show the highest 13–17 Hz power, and low-frequency bursts (LFBs) are low in both. Example from subject 2 across five high-dose propofol infusions. Color indicates feature categorization by an automated algorithm.

To verify this automated categorization, 100 examples of each feature were randomly chosen and presented to an expert human rater. In total, the rater agreed with the script categorization in 283 out of 300 instances: 94% of CBBs, 96% of LFBs, and 93% of spindles. The other 17 examples were deemed ambiguous by the human rater. To illustrate the distinct spectral content of each feature, all examples from subject 2 were graphed comparing spectral power from 3 to 10 Hz and spectral power from 13 to 17 Hz (Figure 2). Activity type, as determined by automated categorization, is shown by color. Three distinct clusters can be identified.

### 3.2. Variation between subjects

**FIGURE 3:**
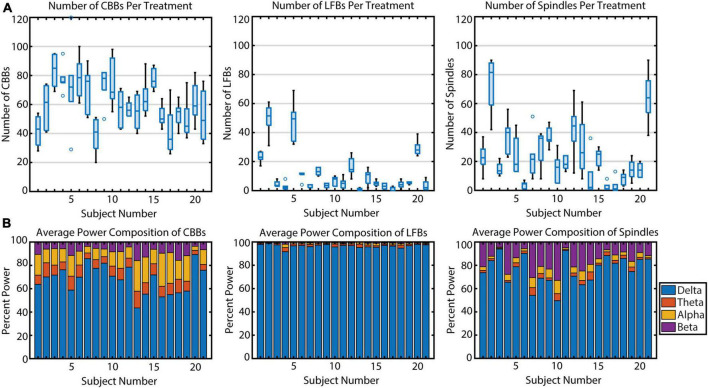
The variation in occurrence and power makeup of all three burst suppression features across 21 subjects. **(A)** The number of features per propofol infusion is shown for each subject. Boxplots indicate median, upper and lower quartiles (blue rectangle), non-outlier range (black), and outliers (blue circles, greater than 1.5 interquartile range outside upper and lower quartiles). **(B)** The relative spectral power composition of each feature type, averaged across propofol infusions, for each subject.

During the propofol infusions, we noticed variability in the types of EEG activity between subjects. The average number of CBBs occurring per subject per infusion ranged from 38.8 to 83.5 (Figure 3A) and was significantly different across subjects (*p* < 0.001, η^2^ = 0.50). Upon further analysis, this variation in CBB occurrence was explained by the following factors: a positive correlation with infusion length (*p* < 0.001, *R*^2^ = 0.80); a negative correlation with average CBSR during the infusion (*p* < 0.001, *R*^2^ = 0.97); a negative correlation with CBB length (*p* < 0.001, *R*^2^ = 0.80); and a marginal effect of sex, with males tending to have more CBBs than females (*p* = 0.053, *R*^2^ = 0.09). The occurrence of LFBs was also quite variable between participants (*p* < 0.001, η^2^ = 0.78), with four subjects exhibiting >20 LFBs per treatment and the other 17 showing few-to-no LFBs (< 20 LFBs per treatment). This variance was explained by a positive correlation with treatment number (*p* = 0.01, *R*^2^ = 0.04), with later infusions containing more LFBs. There was also a significant effect of sex, with females tending to have more LFBs (*p* = 0.027, *R*^2^ = 0.39). Treatment length, average CBSR, and LFB duration were non-significant (*p* > 0.05). Spindle occurrence also had large variation (*p* < 0.001, η^2^ = 0.62), with ten subjects having <20 spindles per treatment, nine subjects having 20–60 spindles per treatment, and two subjects averaging over 60 spindles per treatment. These differences were explained by a positive correlation with average spindle length (*p* < 0.001, *R*^2^ = 0.53), with those subjects who had a higher occurrence of spindles also tending to have a longer duration of spindles. All other parameters were non-significant. In summary, CBB variation between subjects was mostly explained by infusion-related factors (length of infusion, anesthetic depth, length of CBB). LFBs occurred more often in women and more often during later infusions, while spindles occurred more often in those subjects who had longer spindles.

Next, we quantified individual variation in spectral content for the three types of EEG features (Figure 3B). In general, power spectral density follows a 1/frequency distribution (Hooge et al., 1981), so all features are dominated by the lowest frequencies of background noise. The relative power composition differed significantly between features, as expected by the way they were defined. However, the relative power makeup of features also differed significantly across subjects. The percent power in each band (delta, theta, alpha, and beta) differed significantly across participants for all three features (all *p* < 0.001) due to multiple factors (Table 3). For CBBs, increased age and increased CBB duration were significantly correlated with lower relative delta power and higher relative theta, alpha, and beta power. Male sex at birth was significantly associated with higher relative alpha power, and higher BSR was associated with higher beta power. For LFBs, increased age was significantly associated with higher relative alpha power, while sex was not significantly correlated with any relative band power. For spindles, age and sex were not significantly correlated with any relative band power, while increased average BSR was associated with lower relative delta and higher relative beta and alpha, and spindle duration was significantly correlated with lower relative delta power and higher relative beta power.

**TABLE 3:** Factors explaining differences in relative spectral power makeup of CBBs, LFBs, and spindles between subjects.

		Average BIS BSR		Average feature duration		Sex		Age	
		***R* ^2^**	**Dir.**	***R* ^2^**	**Dir.**	***R* ^2^**	**Dir.**	***R* ^2^**	**Dir.**
CBBs	Delta	–	–	0.26***	Neg.	–	–	0.60***	Neg.
	Theta	–	–	0.20***	Pos.	–	–	0.58***	Pos.
	Alpha	–	–	0.20***	Pos.	0.42*	Pos. M	0.45**	Pos.
	Beta	0.08*	Pos.	0.13**	Pos.	–	–	0.59**	Pos.
LFBs	Delta	–	–	–	–	–	–	–	–
	Theta	–	–	–	–	–	–	–	–
	Alpha	–	–	–	–	–	–	0.19*	Pos.
	Beta	–	–	–	–	–	–	–	–
Spindles	Delta	0.09*	Neg.	0.29***	Neg.	–	–	–	–
	Theta	–	–	–	–	–	–	–	–
	Alpha	0.06*	Pos.	–	–	–		–	–
	Beta	0.09*	Pos.	0.34***	Pos.	–	–	–	–

These factors were evaluated using 12 linear mixed models: one for each type of feature and frequency band. Columns indicate *R*^2^ value and direction of correlation (positive, negative, higher for men than women). *P*-values are indicated by symbols and are uncorrected for multiple comparisons. ***p < 0.001, **p < 0.01, *p < 0.05, N.S. (–), *p* > 0.05.

### 3.3. Timing between features

**FIGURE 4:**
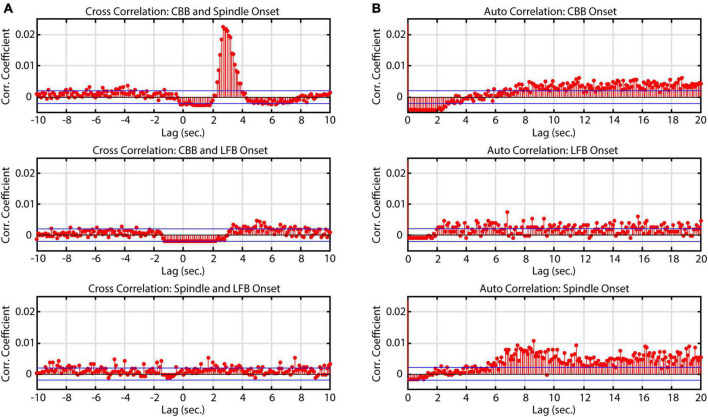
Correlation of onset times between burst suppression feature types. Blue lines denote upper and lower 99% confidence intervals. Time series data consisted of all BIS Monitor burst suppression EEG from all subjects (approximately 2820 min). **(A)** Cross-correlograms between CBB, LFB, and spindle onset. Spindles tend to occur immediately after canonical broadband bursts. **(B)** Auto-correlograms of CBB, LFB, and spindle onset. Spindles were most likely to occur about 8 s after another spindle.

Similarly to Huotari et al. (2004), we noticed that spindles often occurred directly after CBBs but also occurred independently. To evaluate the relationship between features in time, a cross correlation was conducted. Significant correlations were found between CBBs and spindles from 2 to 4 s, confirming the observation that spindles often occur directly after CBBs (Figure 4). Across all subjects, 45% of spindles occurred 2–4 s after a CBB. This ranged from 6.7 to 92% of the spindles from each subject. There was no significant correlation between total number of spindles and number of spindles occurring 2–4 s after CBBs for each subject. No significant trend was seen in the correlations between bursts and LFBs or LFBs and spindles. Autocorrelations were also run on each time series, showing that spindles tended to occur 8 or more seconds after other spindles, while no significant autocorrelation was found for CBBs or LFBs.

### 3.4. Relationship to C_*e*_ and time since bolus

We hypothesized that certain EEG features might be more or less likely to occur at certain effect site concentrations (C_*e*_) or at certain times since infusion start. To evaluate a possible C_*e*_ relationship, C_*e*_ was calculated at the onset of each feature and averaged across each type of feature for each infusion. There was no significant relationship between feature type and C_*e*_. Similarly, the time since bolus was calculated at the onset of each feature and averaged across each type of feature for each treatment. This model showed that there were significant differences in average time of onset for the three features (χ^2^ = 9.66, *p* = 0.008). A Tukey’s HSD analysis showed that spindles occurred significantly later in time than both CBBs (*p* = 0.011) and LFBs (*p* = 0.044) but that there was no significant difference in time of onset between CBBs and LFBs (*p* > 0.05). In summary, the occurrence of spindle activity increased later during infusions; no association was identified between C_*e*_ and EEG features.

### 3.5. Multichannel EEG and spatial variation

**FIGURE 5:**
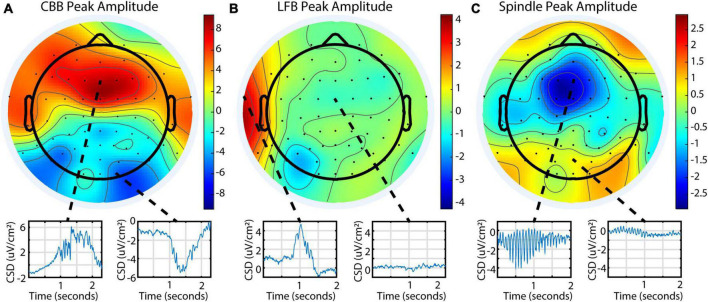
Different burst suppression features show consistently different EEG patterns across the scalp in subject 20 during a single infusion. Data is from the 64-channel EEG of subject 20, infusion 6. Values shown are current source density from a Laplacian transform. **(A)** Canonical broadband bursts exhibit different amplitudes and polarities in an anterior/posterior pattern. **(B)** Low-frequency bursts are localized and primarily detected in the left or right frontotemporal region in this subject. **(C)** Spindles are seen across all electrodes but are mostly negative and largest in amplitude in medial frontal electrodes.

To explore burst suppression variability across the cortex, 64-channel EEG was recorded for a subset of 9 subjects. The EEG was segmented into features using a representative channel, and feature parameters were averaged across the treatment for each electrode. Feature occurrence in this dataset was quite variable, similar to the BIS Monitor dataset: all 9 subjects had consistent CBBs, while 1 subject had prominent LFBs, and 6 had prominent spindles. This was also consistent with the occurrence of features as evaluated from each subject’s BIS Monitor data. Each feature had distinct patterns of localization and peak amplitudes across the cortex (Figure 5). Canonical broadband bursts tended to occur across all electrodes but differ in their amplitude and sign, with mediofrontal electrodes displaying the highest positive amplitude and posterior electrodes showing large negative displacements. In the single subject with consistent LFBs for whom a multichannel EEG recording was available, LFBs were detected in isolated areas across the cortex. Approximately 75% of the LFBs were detected in the left frontotemporal electrodes but seemingly absent in other parts of the cortex. The other 25% were localized to the right frontotemporal electrodes (see Supplementary Figure 2 for raw data examples). All LFBs were primarily positive in amplitude. Spindles were seen consistently across all electrodes and were highest in negative amplitude in mediofrontal areas, with anterior and posterior regions showing smaller positive activity. These patterns were consistent across all subjects measured (Supplementary Figure 3).

### 3.6. BIS Monitor comparison

**FIGURE 6:**
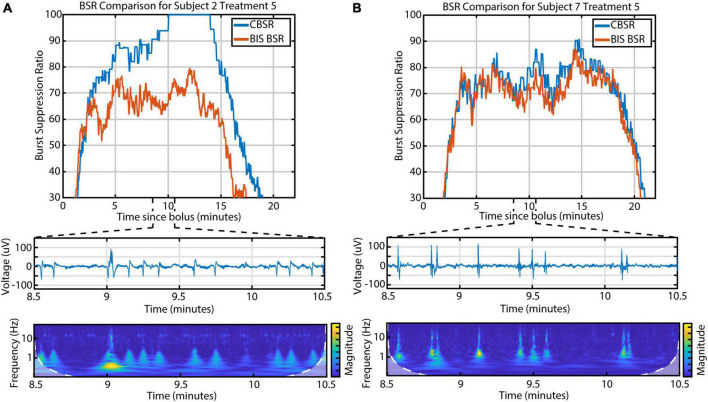
The discrepancy between the BIS BSR and CBSR is largely driven by the occurrence of LFBs and spindles. **(A)** An example infusion with a high occurrence of LFBs, recognizable in both the raw EEG and scalogram, shows a large difference between BSR calculations. **(B)** An example infusion with few-to-no LFBs shows consistency between BSR calculations.

Electroencephalographic monitors are commonly used in clinical settings to evaluate depth of anesthesia in real time. The BIS Monitor in particular processes frontal EEG and calculates a burst suppression ratio (called “BSR”) during deep anesthesia. We found that the BIS Monitor algorithm tends to treat any detectable activity as a burst. This suggests that the BSR is susceptible to artifacts (e.g., movement-related) and that it fails to distinguish the three different kinds of burst activity. We developed a BSR index based only on the presence or absence of CBBs (called “CBSR”) and found that this index diverged from the BIS BSR in participants with a high occurrence of LFBs or high-amplitude spindles (Figure 6). In these subjects, therefore, a fuller characterization of the diversity of EEG activity is necessary in order to completely describe depth of anesthesia during burst suppression.

## 4. Discussion

Though the phenomenon of burst suppression has been appreciated for decades, to our knowledge, no previous studies have systematically and quantitatively examined EEG features and their variability across subjects and across repeated treatments. A thorough description of the diverse manifestations of burst suppression may help explain this unique brain state and its underlying mechanisms. Furthermore, a better understanding of propofol burst suppression has the potential to influence clinical decisions regarding dosing and affect post-operative patient outcomes. Using EEG data obtained during a clinical trial exploring the antidepressant effects of high-dose propofol, we distinguished and parameterized three types of burst-suppression activity: CBBs, LFBs, and spindles. CBBs and spindles have been previously reported, but LFBs appear to be a novel type of activity.

These three types of activity were distinguishable in both the time and frequency domains. CBBs were characterized by long duration, high amplitude, and broad bandwidth, with more alpha and theta power than the other features. LFBs were slightly shorter in duration and had the highest relative delta power. Spindles were the shortest in duration and the smallest in amplitude, with the highest relative beta power. We demonstrated that an automated algorithm was able to segment and categorize the three types of EEG activity, with 94.3% agreement with an expert rater.

### 4.1. The low-frequency burst

Low-frequency bursts appear to represent a previously unappreciated type of neural activity, which is not readily explained as a known EEG feature or technical artifact. LFBs are larger in amplitude and duration than EKG signals, yet smaller than eye blinks and movement artifacts. When compared to sharp waves evoked by painful stimuli, LFBs are longer and lack time-coupling with evoked CBBs (Huotari et al., 2004). The LFB frequency content and intrasubject consistency (across infusions on different days) indicate that it is most likely not caused by electrical interference. Frequent LFBs occurred in subjects both at the beginning and end of the study, indicating they were not brought about by equipment malfunction or other systemic change in protocol. LFBs also do not appear to be CBBs with reduced duration and amplitude, such as those seen during deep hypothermia (Brandon Westover et al., 2015), as they have a markedly different power spectrum. LFBs were captured using both the BIS Monitor and 64-channel EEG in one subject, suggesting they are not hardware- or software-related. Finally, their localized cortical nature differentiates them from many other common EEG artifacts. For further confirmation, data from this study was presented to an expert neurologist with experience examining burst suppression EEG who verified that LFBs do not appear to be artifacts or resemble any known patterns from previous literature. Instead, LFBs resemble neural activity localized to the frontotemporal electrodes. Based on these arguments, low-frequency bursts appear to be a type of genuine brain activity elicited by propofol during the burst suppression state.

In some ways, low-frequency bursts resemble K-complexes: large, negative deflections seen during stage 2 sleep that can occur spontaneously or after a sensory stimulus. The K-complex tends to be approximately 1 s long, consists of high delta power, and can happen locally across the cortex, especially in frontal areas (McCormick et al., 1997; Happe et al., 2002). They are often followed closely by a sleep spindle that is similar to propofol spindles. K-complexes may indicate a suppression of cortical activity in order to continue sleep during non-threatening stimuli (Cash et al., 2009). They have also been compared to sharp waves evoked by painful stimuli of the median nerve during propofol anesthesia (Huotari et al., 2004).

Lewis et al. (2018) showed a similar type of activity, termed “large potentials” (LPs), that occurred after cessation of propofol anesthesia but before consciousness. LPs were high-amplitude, slow (∼1 s) waveforms that occurred most often after salient auditory stimuli. These events seemed to be localized, being detected most strongly in frontal and temporal intracortical electrodes in all epileptic subjects, and they were seen in 4 out of 10 healthy participants in frontal scalp electrodes. The similar morphology and localized occurrence of K-complexes, large potentials, and low-frequency bursts may indicate similar neural mechanisms during three distinct phases of unconsciousness (stage 2 sleep, anesthetic emergence, and anesthetic burst suppression). Though it isn’t clear if LFBs can be intentionally triggered by a sensory stimulus, they could have been triggered by a multitude of auditory or tactile stimuli occurring during propofol infusion. Their inconsistent detection across subjects could be indicative of differential thalamic inhibition by propofol, or it could be due to the inherent limitations of scalp EEG.

The unique spatial and temporal qualities of the low-frequency burst imply that it may have a different generating mechanism than canonical broadband bursts or spindles. The CBB is made up of a slowly varying component (delta wave) overlaid with faster alpha oscillation, while the LFB lacks an alpha component. The alpha component of propofol anesthesia, which persists during burst suppression, is believed to originate from thalamocortical networks (Ching et al., 2010). This frequency distinction, along with the localized nature of LFBs, suggests that they may originate from intracortical activity.

### 4.2. EEG feature variation across subjects

We observed that the occurrence of these features varied across repeated infusions and different subjects. CBB variance was explained by infusion length and anesthetic depth, as expected: longer infusions at lighter anesthesia concentrations tended to have more CBBs. In contrast, LFB and spindle variance was not well explained by these variables. Certain subjects had significantly more LFBs or spindles across all of their infusions, and this was unrelated to infusion parameters. For LFBs, there was a significant correlation with sex, as all four of the subjects who had frequent LFBs were females. However, the other seven female subjects had few-to-no LFBs. This inconsistent occurrence across subjects could be due to the inhomogeneous nature of LFBs across the cortex. Localized bursts have been seen before during both coma (An et al., 2015) and propofol anesthesia (Lewis et al., 2013) and may come about due to the different metabolic thresholds of burst suppression in different neural circuits (Brandon Westover et al., 2015). We observed that propofol has a wide range of effects across subjects even at similar doses: some subjects continue breathing and exhibit spontaneous movement, while others need airway assistance and are completely still. If LFBs only occur in specific areas of the cortex, such as the temporal LFBs seen in one subject’s 64-channel EEG data, perhaps this is due to the differing effects of propofol across the brain. LFBs occurring in non-frontal areas may not be detected by the frontal electrodes of the BIS Monitor. However, this doesn’t explain the inconsistent occurrence across the 9 subjects with 64-channel EEG data. Regarding spindles, the lack of consistency across all subjects may be related to their short duration and low amplitude. With EEG amplitude in general being attenuated by certain factors (thicker skull, increased age), some spindles may occur but are too small to detect when compared to background noise.

Feature occurrence did not vary with estimated effect site concentration, but spindles were more often seen later in the infusion when compared to CBBs and LFBs. The average spindle frequency was 14.4 ± 1.1 Hz, similar to literature which has shown 13–17 Hz spindles during propofol anesthesia. We also noted that the relative spectral makeup of these three feature types differed across subjects. It has been shown that canonical broadband bursts differ in amplitude and spectral power according to subject age (Kratzer et al., 2020). In our data, older subjects with longer CBB duration tended to have CBBs with lower relative delta power and higher relative theta, alpha, and beta power, and males tended to have CBBs with higher relative alpha power. Older subjects also exhibited LFBs with higher relative alpha power. Age and sex did not explain any significant variance in spindle spectral makeup, but longer spindles tended to have lower relative delta and higher relative beta power, and spindles that occurred at high BSRs tended to have lower relative delta and higher relative alpha and beta power.

### 4.3. Spatial patterns across the cortex

In the 64-channel EEG data, each feature displayed a distinctive pattern in amplitude and sign across the scalp, further displaying differences between the three types of electrical activity. Burst suppression has often been described as a homogeneous cortical state, though some researchers have found timing and frequency differences in bursts across the cortex (Lewis et al., 2013). We found that CBBs and spindles seem to occur simultaneously across the scalp, though with differing amplitudes and polarities. In contrast, the LFBs seen in one subject did not occur consistently across the cortex and displayed as distinct localized activity primarily in the left or right frontotemporal electrodes. This data is in line with previous literature and suggests that some burst suppression features may be a global cortical phenomenon while others may be locally limited. We are unable to comment on subdural or subcortical electrical patterns, as our data was limited to scalp EEG.

### 4.4. Burst suppression as a non-binary state

Finally, we examined the effects of these EEG features on the BIS Monitor BSR. An accurate evaluation of anesthetic depth is key in clinical practice, both to avoid patient waking and minimize cognitive aftereffects. Many standard scales, such as the Modified Observer’s Assessment of Alertness and Sedation scale, are coarse and unreliable once the patient stops responding. Thus, the BIS Monitor is a useful tool, especially when evaluating deeply anesthetized patients. However, it has been previously noted that certain frontal EEG monitors underestimate BSR when compared to a human rater (Muhlhofer et al., 2017). Additionally, differing burst morphologies across different anesthesia types could affect accurate monitoring (Fleischmann et al., 2018), and the occurrence of spindles could affect the calculations of the BIS Monitor (Besch et al., 2011). To explore this further, we created a CBSR measurement that only included canonical broadband bursts. We noted a large difference between the BIS BSR and CBSR in certain subjects, especially those with a high occurrence of LFBs and spindles. Although it isn’t clear what causes such variation in EEG activity during burst suppression, there is potentially a difference in anesthetic depth and outcome between a patient with mostly CBBs and a patient with all three burst suppression features.

### 4.5. Limitations

Some limitations of this study include the hardware used and the patient demographic. Due to the nature of the EEG-guided dosing used in this study, the BIS Monitor was required for most infusions. The BIS Monitor is only capable of recording limited frontal EEG; thus, for the majority of burst suppression examples presented here, little can be inferred about spatial patterns across the cortex. There could be additional localized burst suppression features or patterns that are either not detected by frontal EEG or not detectable by scalp EEG overall. More comprehensive 64-channel EEG, including a single recording with LFB occurrences, was only available for one infusion in 9 out of 21 subjects. Therefore, caution must be taken when generalizing these findings, as this data may not fully represent all neural activity during propofol burst suppression. The BIS Monitor is also hardware filtered to exclude frequencies above 45 Hz, so these frequencies could not be explored in the majority of subjects. All treated subjects were actively experiencing a depressive episode, and it is unknown if treatment-resistant depression or common depression medications affect the burst suppression patterns of propofol anesthesia. Finally, burst suppression was only achieved for 15 min of each treatment, so we were unable to explore longer time scales or the effects of extended duration anesthesia. Follow-up studies could explore propofol burst features in a large, healthy population to determine if feature variance affects clinical outcomes such as post-operative cognitive deficits or recovery time. A more varied dose range is also warranted to more thoroughly explore burst features across the entire BSR range.

## 5. Conclusion

Overall, this study demonstrated the non-binary nature of propofol-induced burst suppression and quantified EEG feature variation across repeated infusions in a cohort of subjects with Major Depressive Disorder. Not only are there electrical features beyond the typical bursts and suppressions, but these features vary significantly in their likelihood of occurrence, time and spectral-based parameters, and amplitude patterns across the cortex. EEG activity with large quantities of low-frequency bursts and spindles may not be accurately described by commonly used parameters such as the BIS Monitor burst-suppression ratio. Better quantification of burst suppression types may lead to a better understanding of this phenomenon, more effective dosing techniques, and improved postoperative outcomes.

## Data availability statement

The raw data supporting the conclusions of this article will be made available by the authors, without undue reservation.

## Ethics statement

The studies involving human participants were reviewed and approved by The University of Utah Institutional Review Board. The patients/participants provided their written informed consent to participate in this study.

## Author contributions

BM and ST designed the original clinical trial with contributions from KK. BM, KJ, and KK designed the current study. JJ, AL, DO, and ST performed the propofol infusions. BM, ST, and KJ gathered the data. CL, JH, and KK modeled the propofol effect site concentration. ME helped design the EEG analysis. KJ and SL performed the EEG analysis. KJ performed the statistical analysis and wrote the first draft of the manuscript. KJ, CL, and BM wrote sections of the manuscript. SR examined the data as an expert neurologist and contributed to the discussion section. All authors contributed to the review and editing of the manuscript.
