# Supplementary material for: Diversity of electroencephalographic patterns during propofol-induced burst suppression
Source: Front Syst Neurosci. 2023 Jun 15;17:1172856. doi: 10.3389/fnsys.2023.1172856 (PMC10309040; doi:10.3389/fnsys.2023.1172856)
Supplement: Supplementary file 1 [file Data_Sheet_1.docx]

Supplementary Material

# Supplementary Figures and Tables

## Supplementary Tables

| **Subject** | **Medications at Baseline** | **Changes in Medications** |
| --- | --- | --- |
| **1** | estradiol, citalopram, diazepam, gabapentin, trazodone |  |
| **2** | duloxetine, quetiapine, gabapentin, isotretinoin, aripiprazole, Armour Thyroid |  |
| **3** | lithium, gabapentin, quetiapine, ranitidine, sildenafil |  |
| **4** | L-theanine, estradiol, progesterone, fremanezumab-vfrm, verapamil, sumatriptan, escitalopram, lorazepam, lithium carbonate, imipramine | addition of trazodone after infusion 3 |
| **5** | acetylcysteine, coenzyme Q10, thyroid supplement, cholecalciferol, ferrous sulfate, progesterone |  |
| **6** | carvedilol, aspirin, astaxanthin, biotin, cholecalciferol, ketamine (intranasal), L-methylfolate, lisdexamfetamine, lurasidone, methylcobalamin, omeprazole, coenzyme Q10, bupropion, cariprazine, levothyroxine, pregabalin, carbetalol |  |
| **7** | mixed amphetamine salts, gabapentin, escitalopram, levonorgestrel (intrauterine device) | no mixed amphetamine salts taken on the day of infusion 6 |
| **8** | escitalopram, lamotrigine, gabapentin, buspirone, methylphenidate, fexofenadine |  |
| **9** | (none) |  |
| **10** | meloxicam, cyanocobalamin, lamotrigine, testosterone, albuterol, minocycline, famotidine, omeprazole, tretinoin, hydroxyzine, desonide |  |
| **11** | bupropion, sumatriptan, zolpidem, aripiprazole, mupirocin, duloxetine, clonazepam |  |
| **12** | trazodone, mometasone, ondansetron, bupropion, clonazepam, levothyroxine, hydroxychloroquin, naproxen |  |
| **13** | cyanocobalamin, atorvastatin, cetirizine, pantoprazole, lamotrigine, methylphenidate, bupropion, desvenlafaxine, meloxicam | aripiprazole added before infusion 2 |
| **14** | albuterol, hydroxyzine, testosterone, valcyclovir, loratadine, bupropion, desvenlaflaxine, topiramate |  |
| **15** | mupirocin, azelastine, albuterol, fluocinonide (topical), emtricitabine-tenofovir, sildenafil, bupropion, alprazolam, testosterone |  |
| **16** | bupropion, desvenlafaxine, aripiprazole |  |
| **17** | zolpidem, alprazolam, bupropion, duloxetine, mirtazapine, oxybutynin, acyclovir |  |
| **18** | budesonide-formoterol, ondanestron, diazepam, buspirone, azelastine, omeprazole, cetirizine, naphazoline-pheniramine |  |
| **19** | levomilnacipran, amphetamine-dextroamphetamine, aripiprazole, bisoprolol, bupropion, buspirone |  |
| **20** | escitalopram, famotidine, norethindrone acetate and ethinyl estradiol, pantoprazole |  |
| **21** | desvenlafaxine, modafinil, desmopressin |  |

**Supplementary Table 1:** A list of medications each subject was taking before the propofol infusions. All medications were continued unchanged throughout the six infusions unless indicated under “Changes in Medications”.

## Supplementary Figures


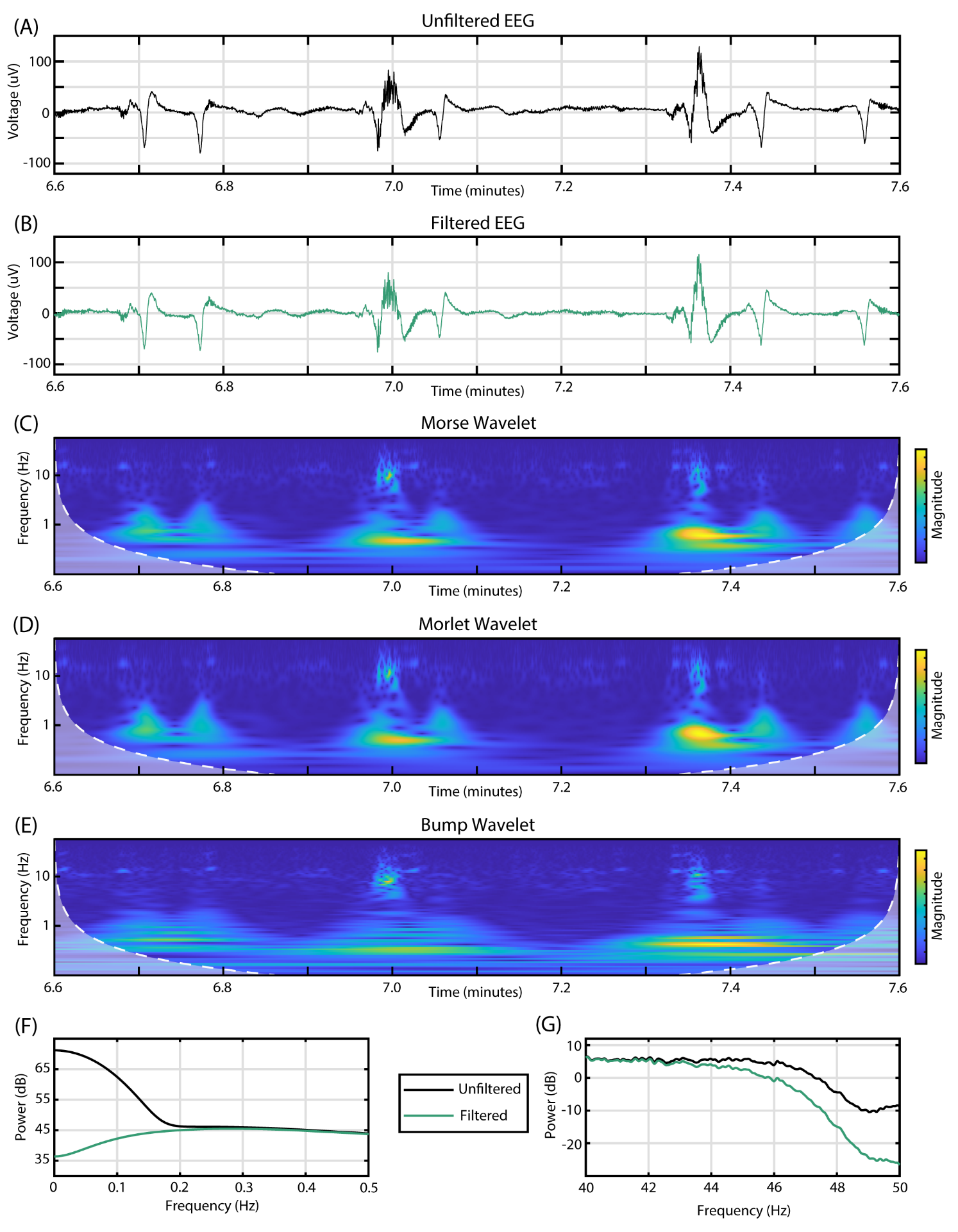


**Supplementary Figure 1:** An illustration of the processing steps and possible alternatives for the BIS Monitor EEG data. Data is from subject 2, infusion 5. **(A)** The raw EEG imported from the BIS monitor (DC offset manually subtracted, converted to μV using a coefficient of 0.05). **(B)** The EEG signal after low- and high-pass filtering. **(C)** The scalogram of the signal in (B) computed using the Morse wavelet. **(D)** The same scalogram, but computed using the Morlet wavelet, or the **(E)** bump wavelet. **(F)** The frequency response of the filtered and unfiltered signals; note that they are similar until < 0.2 Hz. **(G)** The frequency response of both signals from 40-50 Hz. Note that the unfiltered signal starts declining at 45 Hz, possibly because of a hardware filter in the BIS Monitor.


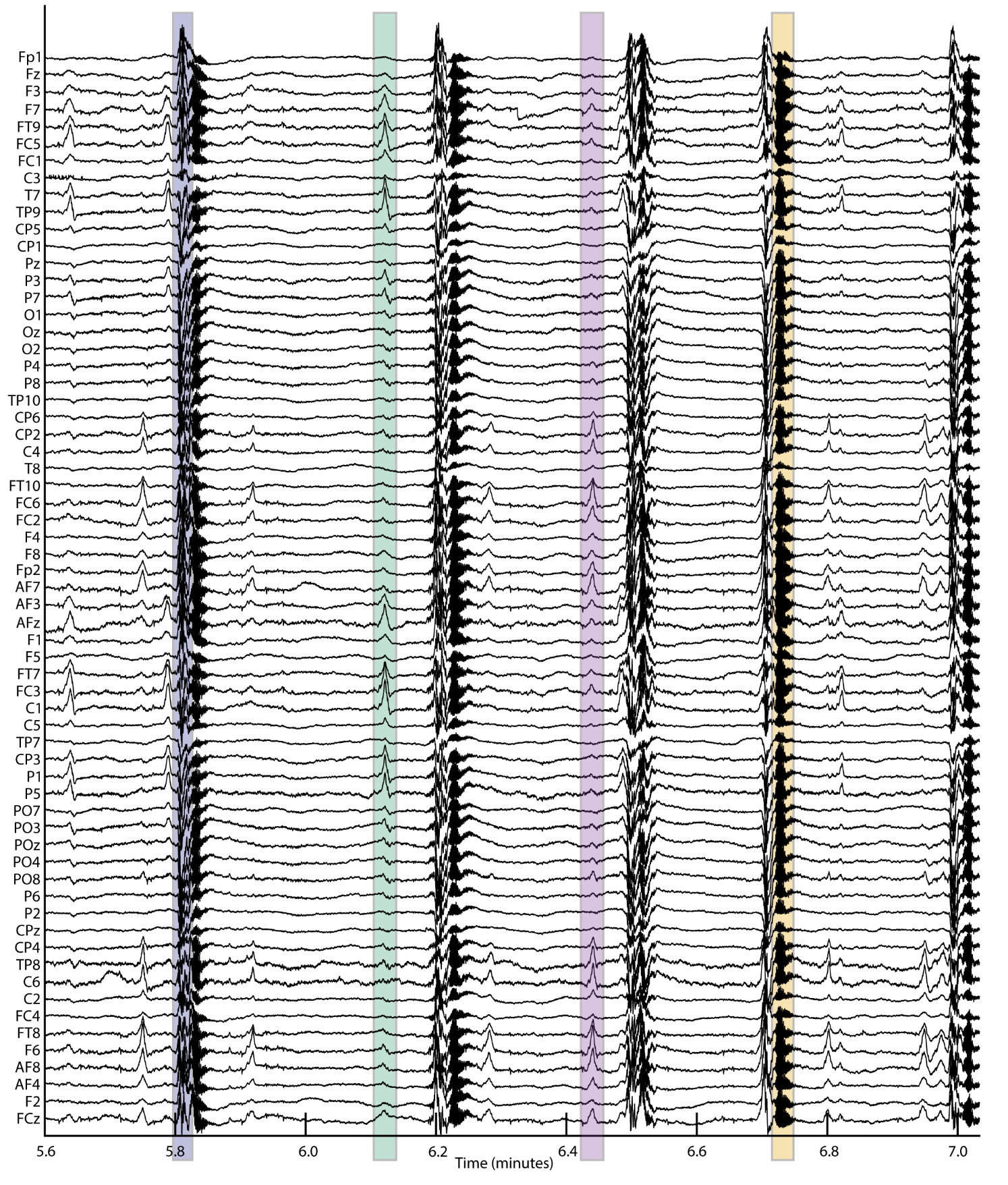


**Supplementary Figure 2:** An example of 64-channel EEG data from subject 20. Data with a reference of Cz was used for clarity. Several co-occurring EEG features have been colored, including a canonical broadband burst (blue), a low-frequency burst localized to the left temporal cortex (green), a low-frequency burst localized to the right temporal cortex (purple), and a spindle (yellow).

##
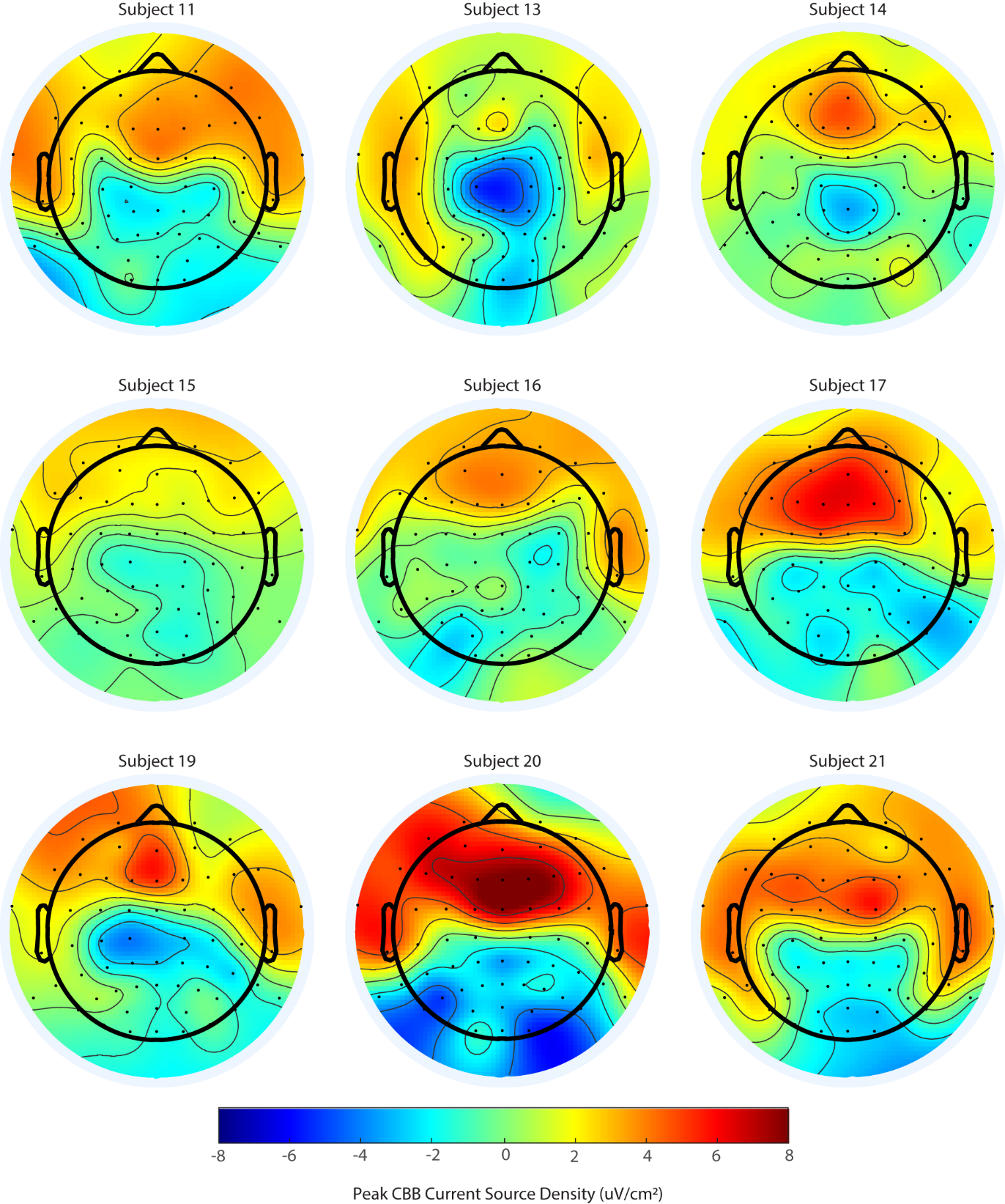


## Supplementary Figure 3: Peak current source density values from 9 subjects, one infusion each. Peak values were averaged across all CBBs at each electrode.
